# Age, period, and cohort trends of substance poisoning, alcohol-related disease, and suicide deaths in Australia, 1980–2019

**DOI:** 10.1007/s00127-024-02749-4

**Published:** 2024-08-23

**Authors:** Chrianna Bharat, Ria E. Hopkins, Mark Chambers, Louisa Degenhardt, Amy Peacock, Michael Farrell, Wing See Yuen, Nicola Man, Natasa Gisev

**Affiliations:** 1 National Drug and Alcohol Research Centre, UNSW Sydney, 22-32 King St, Randwick, Sydney, NSW 2031 Australia; 2 School of Psychology, University of Tasmania, Hobart, Australia

**Keywords:** Suicide, Drug overdose, Alcohol-related disorders, Mental health, Illicit drugs

## Abstract

**Purpose:**

Deaths due to substance poisoning, alcohol-related disease, and suicide pose a critical public health issue, and have been categorized as “deaths of despair” in the US. Whether these deaths represent a distinct phenomenon requires exploration, particularly in other countries.

**Methods:**

This retrospective observational study examines age-period-cohort trends of (combined and cause-specific) substance poisoning, alcohol-related disease, and suicide deaths among Australians aged ≥15-years that occurred between 1980 and 2019 and compares trends between males and females.

**Results:**

Combined mortality rates were initially (1980–1999) relatively stable, reflecting a reduction in alcohol-related disease deaths offset by an increase in substance poisoning deaths. A decline (2000–2006) and subsequent increase (2007–2019) in combined rates were primarily attributable to corresponding changes in both substance poisoning and suicide deaths among males. Distinct age-period-cohort trends were observed between cause of death sub-types, with net drifts: increasing for male (net drift [95% CI]: 3.33 [2.84, 3.83]) and female (2.58 [2.18, 2.98]) substance poisoning deaths; decreasing among male alcohol-related disease (− 1.46 [− 1.75, − 1.16]) and suicide deaths (− 0.52[− 0.69, − 0.36]); and remaining relatively stable for female alcohol-related disease (− 0.28 [− 0.66, 0.09]) and suicide deaths (− 0.25 [− 0.52, 0.01]).

**Conclusions:**

Although combined age-specific trends were relatively stable over the study period, different and distinct patterns were observed within cause-specific deaths, challenging the notion that these causes of death represent a distinct epidemiological phenomenon. These data indicate a critical need to review the appropriateness of guidance for clinical practice, prevention strategies, and policy initiatives aimed at preventing future deaths.

**Supplementary Information:**

The online version contains supplementary material available at 10.1007/s00127-024-02749-4.

## Introduction

Deaths due to substance poisoning, alcohol-related disease, and suicide pose a critical public health issue, and have been grouped and described by some as “deaths of despair”, referring to the notion that they stem, in large part, from a process of underlying, intersectional, and cumulative disadvantage [1, 2]. Although these deaths can have overlapping mechanisms (e.g., drug poisoning is a common mechanism of suicide [3, 4]), the question of whether they represent a distinct epidemiological phenomenon with the same underlying driver/s remains the subject of contentious debate.

The seminal works by Case and Deaton reported that deaths due to substance poisonings, alcohol-related liver disease, and suicide were responsible, in large part, for the rise in mid-life mortality and decreasing life expectancy in the United States (US) [1, 2]. Cumulative disadvantage, resulting from population-level declines in physical and mental health, and labour market opportunities, was theorised as a primary driver of increasing distress. In turn, this was thought to have led people to engage in activities and/or behaviours resulting in premature death [1, 2]. Although plausible, without data on mental health assessments, the implication that these trends are the result of individual suffering is unqualified. Other analyses of US data have observed that trends of these three causes of death are notably different from one another, further challenging the notion of a common underlying driver [5].

The term “deaths of despair” has been used in the literature to embody a range of often overlapping concepts. The original analysis considered deaths due to suicide; substance poisonings (including alcohol); and alcohol-related chronic liver disease [1, 2]. In subsequent studies, these categories were expanded to capture mental disorders and other diseases explicitly related to drug and alcohol use [6–8]. Variation also relates to whether deaths from the same substance are separated according to whether the mechanism was acute or chronic (e.g., alcohol poisoning grouped as a substance poisoning versus alcohol-related disease). For this reason, between-study and between-country comparisons can be complicated.

Nevertheless, the past decade has seen a rapid increase in the number of studies investigating these specific deaths in tandem, with studies primarily from the US, United Kingdom (UK), and Canada [6, 9–13]. Australian researchers recently applied spatiotemporal techniques to examine deaths due to suicide, substance poisonings, alcohol-related liver disease, and a category capturing ‘all other causes’ [14]. They observed differences in spatiotemporal determinants and found deaths from all other causes were a better predictor of suicide deaths than drug poisoning or alcohol-related liver disease deaths, suggesting a lack of population-level drivers. To better understand the trends in, and historical contexts behind, substance poisoning, alcohol-related disease, and suicide deaths in the Australian context, a detailed side-by-side examination of their evolution as a function of age, period, and cohort effects is required. This will be useful for studies aimed at identifying modifiable determinants and persons at greatest risk, whilst simultaneously examining the utility of “deaths of despair” as a single unitary construct. We expect that, if these deaths comprise a conceptually-related phenomenon, this might be demonstrated by similar patterns for each of the three causes of death.

Accordingly, we examined deaths due to substance poisoning, alcohol-related disease, and suicide among Australians aged ≥15-years between 1980 and 2019. An age period cohort (APC) approach was used to describe the relationship between these deaths and attained age, calendar time (i.e., period) and birth cohort. The specific objectives were to:

1. Assess temporal trends in rates of deaths, overall, and by cause of death (substance poisoning, alcohol-related disease, suicide);
2. Compare sex-specific temporal trends in rates of these deaths; and
3. Examine the age, period, and cohort effects of these deaths.

## Methods

### Data source

The Cause of Death Unit Record File is an Australian national database compiling death records from each of the Australian State and Territory Registries of Births, Deaths, and Marriages, and the National Coronial Information System. Records contain an underlying cause of death, defined as (a) ‘*the disease or injury that initiated the train of events leading directly to death*’, or (b) ‘*the circumstances of the accident or violence that produced the fatal injury*’ [15]. Data were obtained for all deaths between 1980 and 2019 for decedents aged ≥15-years. Due to delays in finalising deaths referred for coronial investigation, data for 2019 were subject to potential future revision at time of analysis. Ethical approval and a waiver of consent to access national death records was provided by the University of New South Wales Human Research Ethics Committee (HC220754).

### Procedure

ICD codes were used to identify deaths for which the underlying cause was attributable to a substance (including alcohol) poisoning, alcohol-related disease, or suicide (Appendix 1). Prior to 1997, causes of death were coded according to the ICD-9; from 1997 to 2019, causes of death were coded according to ICD-10 [7]. Cause of death categories were guided by definitions proposed in the literature, with minor adjustments made to account for variation in Australian coding practices, with a focus on creating homogenous, mutually exclusive groups (Panel 1). Extracted data included age at death, date of death, and sex.

**Panel 1:**
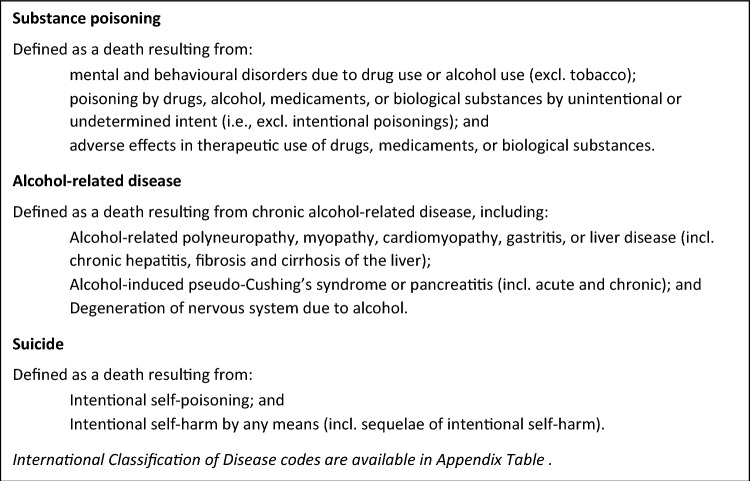
Cause of death definitions

### Statistical analysis

#### Age-standardised mortality rates

Age-standardised mortality rates were calculated for combined and cause-specific (substance poisoning, alcohol-related disease, suicide) deaths, overall and by sex, using a direct age adjustment approach [16]. The reference population was the population structure of Australians aged ≥15-years in 2001. Populations were partitioned into 14 five-year age classes (15–19, 20–24, …, 80–84) with a 15th class accounting for people aged ≥85-years, in accordance with the approach used by the Australian Institute of Health and Welfare. Using notation described by Selvin [16], ${P}_{i}$ denotes the population size of the ${i}^{th}$ age class in 2001 and ${r}_{ij}$ denotes the observed (age class specific) mortality rate of the ${i}^{th}$ age class in year $j$ in deaths per 100,000 person years (PY). Thus, the age-standardised mortality rate in year $j$, ${r}_{j}$, deaths per 100,000 PY is defined as

$${r}_{j}=\frac{\sum_{i=1}^{15}{r}_{ij}{P}_{i}}{P} ,$$

where $P$ is the total population aged ≥15-years in 2001.

#### Age, period, cohort analyses

A statistical APC framework was used to investigate interrelated patterns of age at time of death (age), year of death (period), and year of birth (cohort) [17]. Six categories were analysed, defined by sex and cause of death (substance poisoning, alcohol-related disease, suicide), as well as the combined cause of death group by sex (for a total of eight fitted APC models). For the APC models, deaths were classified by two-year age categories (15–16, 17–18, …, 99+) and two-year period categories (1980–81, 1982–83, …, 2018–19). For each combination of sex and cause of death, the number of deaths occurring in each age-class and period were summed and arranged as a 43 × 20 matrix, as were total PY for the corresponding population at risk. Pairs of such matrices were input into the fitted APC models [18]. Models were fitted using code published by the National Cancer Institute [17], which produced outputs including overall annual percentage change (net drift), estimates of the age-specific annual percentage change (local drifts), and cohort rate ratios.

Analyses were undertaken using SAS v9.4 (SAS Institute Inc., Cary, NC) and R (R Core Team, Vienna) using the *apc* and *hexamap* packages [19]. The study is reported following RECORD guidelines [20].

### Role of the funding source

Funders had no role in study design; collection, analysis, or interpretation of data; writing of the report; or decision to submit this article.

## Results

### Temporal trends

**Fig. 1:**
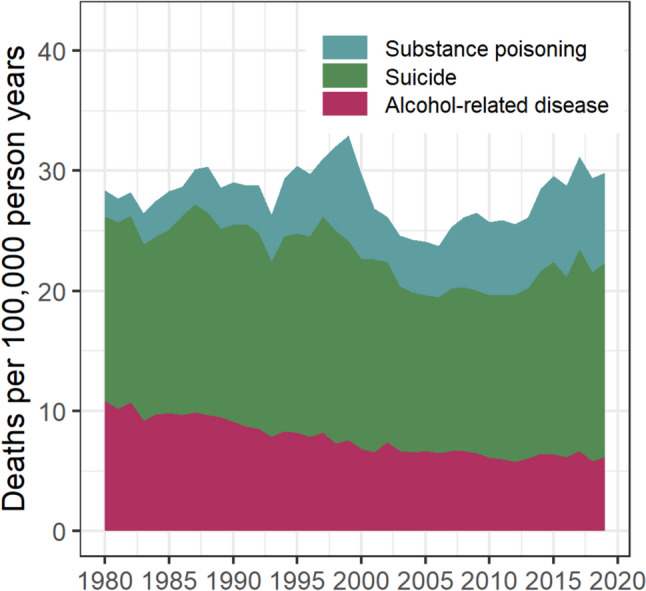
Age-standardised rates of substance poisoning, alcohol-related disease, and suicide deaths, 1980–2019

Figure 1 shows combined and cause-specific mortality rates for deaths due to substance poisoning, alcohol-related disease, and suicide, from 1980 to 2019 (Appendix 2, raw data).

Between 1980 and 1999, combined death rates increased gradually (+16.2%). However, examining cause-specific rates show this period was reflective of both a reduction in alcohol-related disease deaths (from 10.9 to 7.6 per 100,000 PY), and an increase in substance poisoning deaths (from 2.2 to 8.8 per 100,000 PY). After peaking around the late 1990s, combined mortality rates declined by 28.0% (33.0 to 23.8 per 100,000 PY) between 1999 and 2006. This reduction was primarily attributable to a decline in substance poisoning deaths (− 51.8%), although reductions were also observed for alcohol-related disease and suicide deaths (− 14.6% and − 21.5%, respectively). Between 2006 and 2019, combined mortality rates steadily increased (+25.8%), approaching peak rates observed pre-2000. In all study years, suicide was the most common of the three causes: in the most recent calendar year examined (2019), the age-standardised mortality rate for suicide deaths was 16.2 per 100,000 PY, followed by substance poisoning deaths (7.5 per 100,000 PY) and alcohol-related disease deaths (6.2 per 100,000 PY).

### Sex-specific temporal trends

**Fig. 2:**
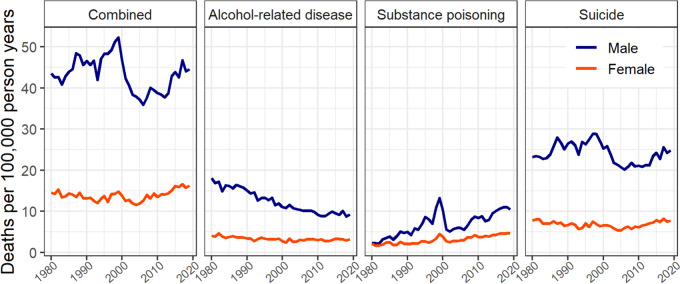
Age-standardised rates of combined and cause-specific (alcohol-related disease, substance poisoning, and suicide) deaths by sex, 1980–2019

Sex-specific mortality rates varied by cause of death and calendar time but remained consistently higher among males (Fig. 2, Appendix 3).

For alcohol-related disease deaths, the absolute difference between sexes was largest in 1980 (age-standardised rate: males, 18.1; females, 4.0) and reduced over time, primarily due to decreasing rates of these deaths among males (2019 age-standardised rate: males, 9.2; females, 3.2). For substance poisoning deaths, the difference in rates between sexes increased over time. While negligible in 1980 (age-standardised rate: 2.2 males; 2.1 females), the rate of substance poisoning deaths in 2019 was twice as high among males compared to females (age-standardised rate: 10.5 males; 4.7 females). These sex-specific investigations also reveal that the decline in the combined rate around the year 2000 was primarily attributable to reduced substance poisoning and suicide deaths among males.

### Age, period, cohort estimates

**Fig. 3:**
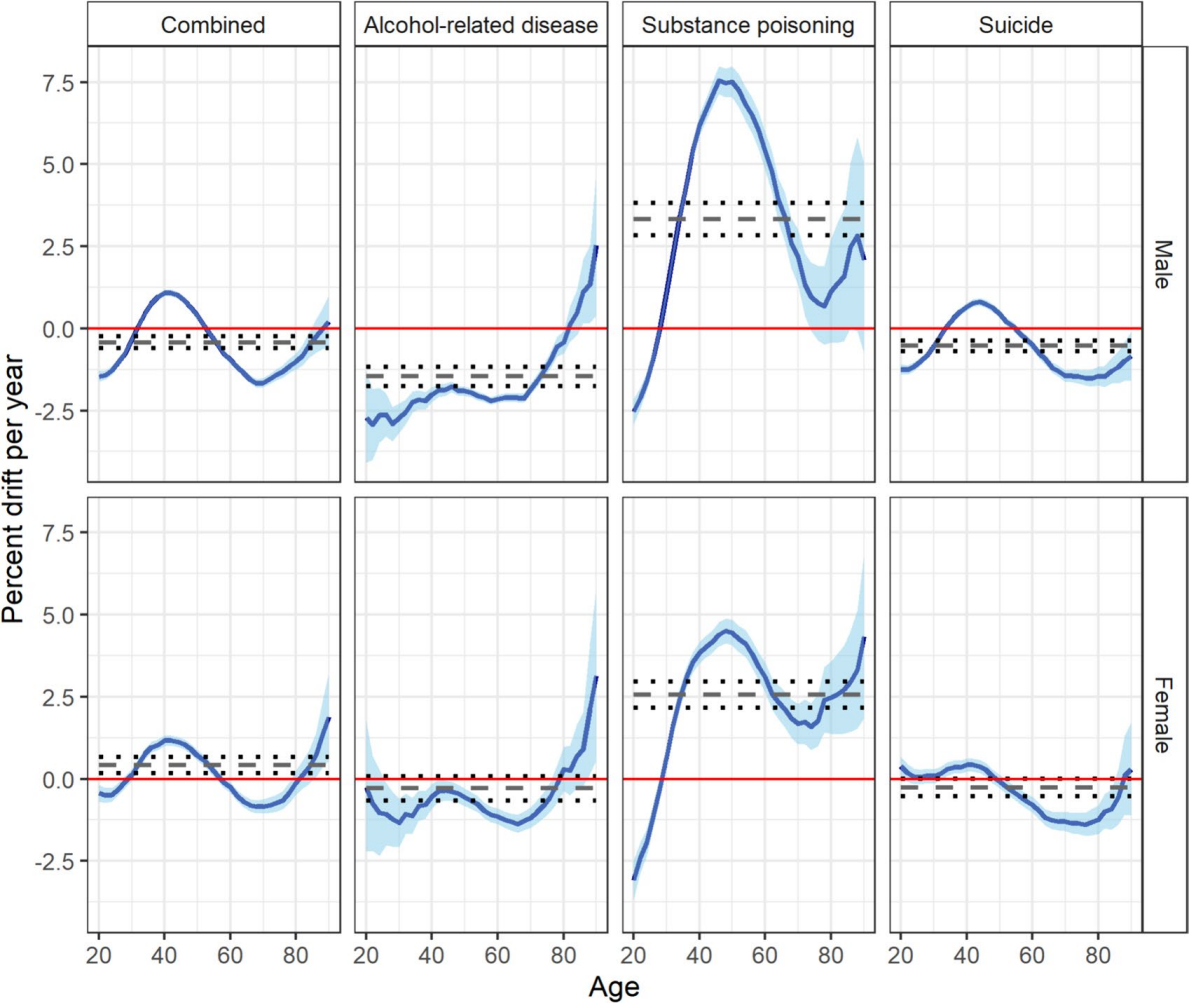
Age-specific annual percentage change of rates of combined and cause-specific (alcohol-related diseases, substance poisoning, and suicide) deaths, by sex, between 1980 and 2019. Dashed horizontal lines are annual percentage changes averaged over all age classes (net drifts) with 95 percent confidence intervals given by dotted lines

Figure 3 demonstrates the overall (net drift) and age-specific (local drift) annual percentage change in rates of combined and cause-specific deaths, stratified by sex (Appendix 4).

**Fig. 4:**
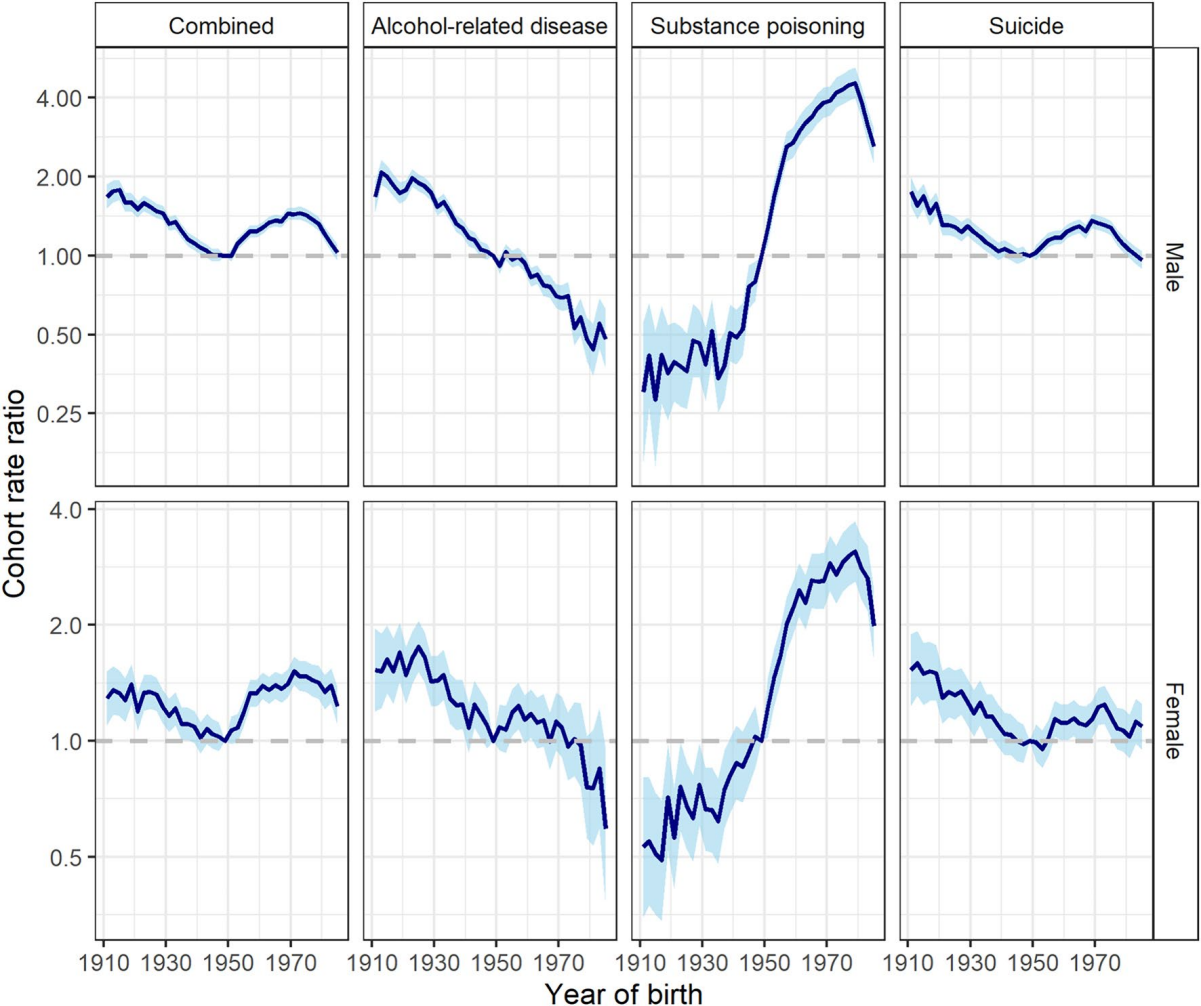
Birth-cohort rate ratios (reference year = 1950) for combined and cause-specific (alcohol-related disease, substance-poisoning, and suicide) deaths by sex

Over the study period, the net drift [95% CI] for combined mortality rates were negligible (males: − 0.41 [− 0.59, − 0.24]; females: 0.42 [0.17, 0.68]). However, these findings represent the net result of substance poisoning, alcohol-related disease, and suicide deaths, each of which demonstrated qualitatively different age and cohort patterns. Figure 4 shows the (birth) cohort rate ratios, stratified by sex.

For substance poisoning deaths, net drifts for males (3.33 [2.84, 3.83]) and females (2.58 [2.18, 2.98]) were substantial, with cohort rate ratios indicating these deaths increased steadily between 1940 to 1980 birth cohorts. Rates decreased among males and females born after 1980, although these remained higher than their counterparts born before the mid-1950s (Figs. 3, 4). These cohort trends contributed to the striking pattern of local drifts; over time, there were negligible changes in substance poisoning death rates among males aged 30-years, compared to a drift of +7.55% per year among males aged 46-years (Appendix 4). Trends in substance poisoning deaths among females followed a similar pattern but at a lower magnitude, with a peak drift of +4.51% per year among females aged 48-years. For male and female Australians aged≤ 30-years, rates of substance poisoning deaths tended to decrease over time.

For alcohol-related disease deaths, sex-specific differences were observed. Birth cohort rate ratios for alcohol-related disease deaths among females were small and resulted in an overall net drift of − 0.28% (95% CI: − 0.66, 0.09). For males, birth cohort rate ratios suggest a decreasing trend in alcohol-related disease deaths in recent birth cohorts. This was further reflected by the negative drift in alcohol-related disease deaths over time across most ages (net drift [95% CI]: − 1.46 [− 1.75, − 1.16]). For both males and females, there was evidence to suggest that alcohol-related disease death rates were increasing over time among those aged ≥80-years, though confidence intervals for the rate ratios are relatively wide.

Rates of suicide deaths declined among both male and female cohorts born between 1910 and 1950 but have been increasing among (primarily male) cohorts born before 1970. The absence of persistent cohort trends in either males or females is further reflected in small (males: − 0.52 [− 0.69, − 0.36]) to negligible (females: − 0.25 [− 0.52, 0.01]) overall net drifts. Local drifts suggest suicide mortality rates have been increasing among males aged 35–50-years (peak local drift at 44-years: 0.81 [0.69, 0.92]), and declining among Australians aged ≥50-years.

**Fig. 5:**
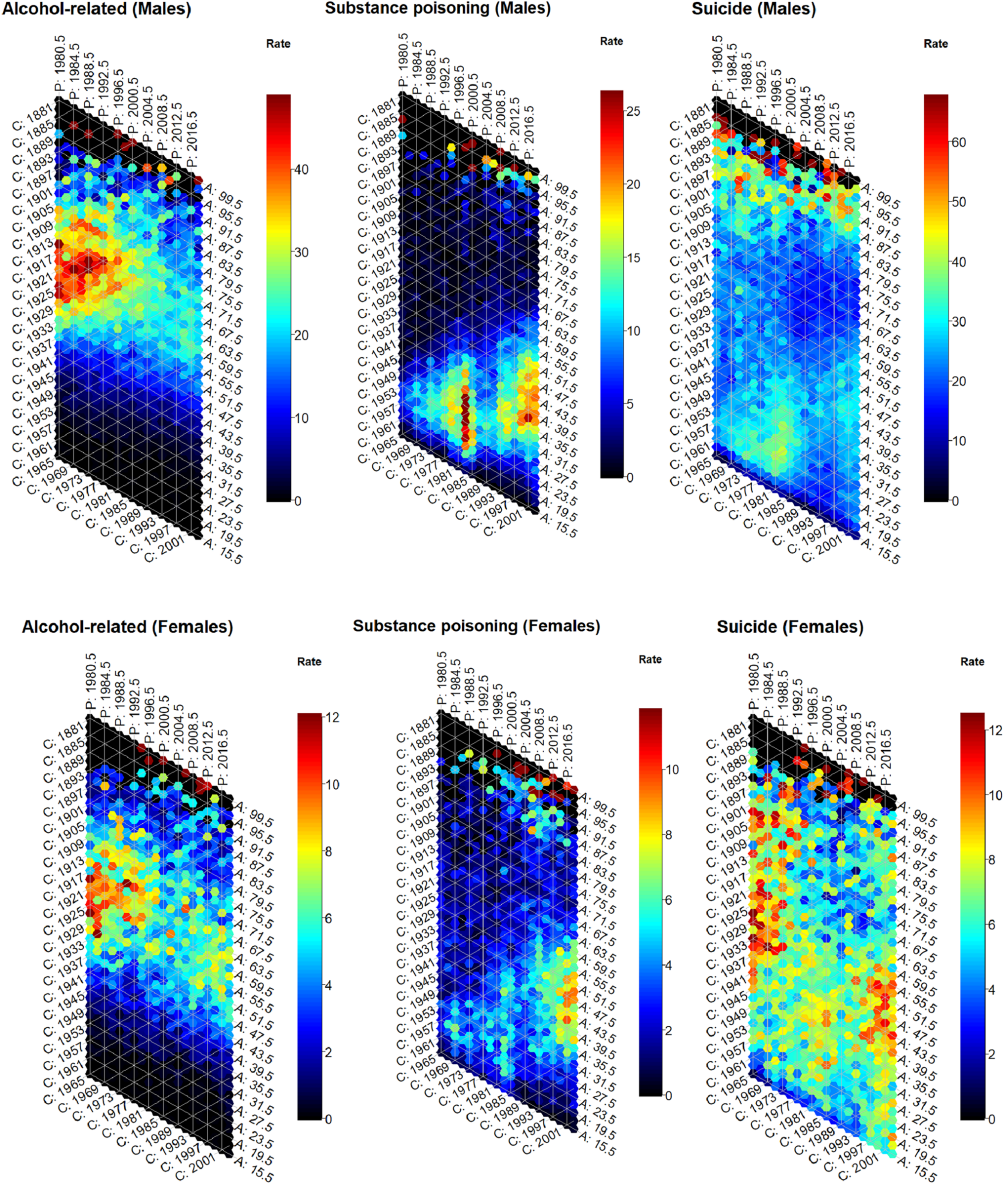
Mortality rates for alcohol-related disease, substance poisoning, and suicide by sex with cohort (C), period (P) and age (A) axes

Age and generational trends are visualised in hexagonal heatmaps showing the intersection of age, period, and cohort (Fig. 5). The diagonal bands of colour for substance poisoning deaths, particularly among males, reinforces the large relative differences between cohorts, as well as the pronounced increase, and subsequent decrease, of these deaths around the year 2000. These figures also suggest rates of substance poisoning deaths among young-middle-aged females were highest towards the end of the study period (2014–2019). The orange/red bands for alcohol-related disease deaths characterise the strongly age-dependent nature of this death subtype, with mortality rates highest among people aged between 50 and 80 years. These plots also reiterate the differences in mortality rates for alcohol-related disease deaths between cohorts, with the highest mortality rates observed among males and females who were in their late 50s and 60s in 1980 (i.e., born around 1920). These visualisations further demonstrate the uniformity in suicide mortality rates among females across ages, periods, and cohorts, and the differences in male suicide mortality rates between cohorts, with patterns of birth cohorts similar to those apparent in substance poisoning deaths.

## Discussion

In this analysis of forty years of Australian mortality data examining deaths due to substance poisoning, alcohol-related disease, and suicide, the most common of the three causes of death over the study period was suicide. Between 1980 and 1999, combined mortality rates were relatively stable, reflecting the combined effect of both a reduction in alcohol-related disease deaths and increase in substance poisoning deaths. This was followed by a decline until 2006 and a subsequent increase to the end of the study period, primarily attributable to corresponding changes in the rates of substance poisoning and suicide deaths among males. Over the study period, combined age-specific mortality rates remained relatively stable, but patterns varied between cause of deaths, as well as by sex, age, birth cohort, and period.

Increases in substance poisoning deaths were of considerable concern in Australia in the late 1990s, largely attributed to increased availability of cheap, higher purity heroin [21]. After the turn of the century, an unprecedented reduction in heroin supply across all Australian jurisdictions was accompanied by a reduction in fatal overdoses [21, 22]. The findings from the present study reflect these period effects of substance poisoning deaths, highlighting the importance of considering changes in the local context—whether they be environmental, social, economic, behavioural, or healthcare-related—when interpreting temporal patterns of death.

In the US, Scotland, and Canada [12], recent and substantial increases in substance poisoning deaths have been attributed in part to increasing prescription medicines use (particularly opioids) [23–25], novel benzodiazepines [26], and increasing use of heroin and illicit fentanyl in North America [23]. This trend is somewhat reflected in the Australian setting, which so far lacks the proliferation of illicit fentanyl, with the increase in substance poisoning deaths since 2006 coinciding with substantial increases in opioid analgesic prescribing [27, 28]. Importantly, changes in administrative coding around this time may have influenced when the observed increase in substance poisonings occurred [15]. Although examining specific drugs involved was beyond the study’s scope, a 2021 analysis showed opioids were the most commonly involved drug class in Australian overdose deaths, with over two-thirds of opioid overdose deaths involving pharmaceutical opioids [29]. Harm-reduction efforts, including drug treatment policies, are important strategies to reduce the scale of these substance-related harms.

Alcohol consumption in Australia increased post-World War II and peaked in the 1970s, attributed to increasing affluence, reduced restrictions, and declines in “puritanism” around this time [30]. In the 1980s, consumption declined and has remained relatively stable since the 1990s. Previous analyses have shown alcohol use tends to peak between the ages of 40–60-years [31], but that alcohol use and risky drinking behaviours have declined among recent birth cohorts [31, 32]. In the present study, these findings are reflected in the older ages and strong cohort trends of alcohol-related disease deaths, whereby rates were highest among 50–80-year-olds across the study period and absolute rates have declined among successive birth cohorts; similar cohort and age trends have been observed internationally [6, 12]. It is unsurprising these deaths appear to occur at older ages: alcohol-related disease mortality captures a range of health effects and chronic diseases resulting from long periods of heavy alcohol consumption [33]. Accordingly, a higher proportion of alcohol-related disease deaths will occur after long induction periods, compared to the more acute poisoning and suicide deaths. Also of note is the difference between sexes, with more substantial declines in alcohol-related disease deaths observed among male compared to female cohorts born after the 1950s. This convergence in male–female alcohol-related disease deaths may reflect the increase in women’s drinking over this time, as well as the narrowing of the gender gap in risky drinking behaviours [34, 35]. Future research will be required to assess whether changes in alcohol consumption among later birth cohorts translate into a greater convergence of alcohol-related disease mortality as these individuals reach mid-life.

Of the cause-specific deaths considered, suicide remained the dominant cause over the study period, with patterns of deaths varying by sex and age. Suicide deaths among older-aged men were consistently higher than among middle-aged men, concordant with prior global research [36]. In the 1990s, the increased rate of suicide deaths among young (20–35-years) males may have related to increasing unemployment in this age group [37], while reduced suicide mortality rates among middle-aged (40–60-years) females may reflect disproportionate antidepressant uptake in this age group [38]. As the circumstances surrounding substance-related deaths can be complex, it is possible that some suicide deaths involving drugs may have been misclassifications (whereby intent was incorrectly deemed intentional), especially considering these deaths coincided with the previously mentioned increase in heroin availability. However, hanging was the most common method of suicide among young men in the 1990s [39], limiting misclassification of poisoning deaths as suicides as an explanation for observed increases in young men. Reasons for the dynamic changes in female suicide deaths over time, particularly the increased rate since 2000, is unclear, and requires further exploration. Regardless of the contributing factors, that suicide deaths remained high throughout the study period demonstrates a critical need to review the effectiveness of current suicide prevention strategies.

Overall, patterns of deaths due to substance poisoning, alcohol-related disease, and suicide differed, providing further evidence that caution is needed when considering a narrative that groups these deaths as driven by shared phenomena [40]. In US and UK studies, as in this study, drug-related deaths appear to be the main driver of increases in overall rates of the collective group [5, 7, 9, 12]. This observation has led researchers to question whether these deaths are indicative of a “despair” problem or a drug overdose problem (or more specifically, an opioid overdose problem) [41, 42]. Given the overlapping and intersectional drivers of these deaths, including socioeconomic disadvantage, declining social support structures, increasing global insecurity—all contributing to rising levels of psychological distress, there remains value in considering these deaths as having shared influences and associated risks. However, our findings add to the growing body of evidence demonstrating the importance for researchers, policymakers, and other stakeholders to remain aware of the heterogeneity between these causes of death, and the potential need for the development of differing strategies to address and prevent these deaths. Our study, and others examining the age, period, and/or cohort effects of these deaths [6, 43, 44], also demonstrate how causes of death differentially affect sub-populations, as well as the effects of local phenomena, such as the impact of disruption to Australian drug markets on substance poisoning deaths. Although strategies targeting underlying drivers and psychological distress may be universal, knowledge of these differences and contexts is essential for the development of targeted and context-specific interventions, as well as the accurate reporting of temporal changes in mortality rates.

APC modelling provides a powerful framework for inferring meaningful structure from complex data. Use of an expanded definition of these deaths allowed the capture of drug and other alcohol-related disease deaths which have the same underlying socio-structural drivers, but which may be excluded by analyses limited to drug poisonings and alcohol-related liver disease. The availability of forty years of mortality data provided scope for examining trends across a period of considerable social and economic change, allowing cohort effects to be examined.

Several limitations must be noted. This study examined cause of death records and may be limited by incorrect or unclear coding. Since 2006, the Australian Bureau of Statistics has implemented a revisions process for improving the quality of cause of death data and, in particular, the coding of intent [15]. This may influence comparisons across calendar time; however, as fewer substance poisoning deaths with undetermined intent (ICD codes Y10-Y15) were observed in the years before compared to after implementation of the revision process (data not shown), the impact of this administrative change on observed patterns is expected to be minimal. Nevertheless, intent may be difficult to ascertain and the underreporting of suicide deaths is a known problem [45]. Although international research has demonstrated associations between these causes of death and socioeconomic factors (e.g., ethnicity, education, employment) [1, 2, 11], detailed data on these were unavailable, and these relationships require further exploration in the Australian context.

## Conclusion

Although combined age-specific trends in deaths due to substance poisoning, alcohol-related disease, and suicide were relatively stable over the last forty years in Australia, different and distinct patterns were observed between these three cause of death categories. These findings highlight the importance of considering the local context in interpreting mortality trends and how it may influence the mechanisms and means by which these harms occur. These findings also indicate there is a critical need to review the appropriateness of considering these causes of death as a distinct phenomenon, as well as an ongoing need to review the effectiveness of current policies and strategies aimed at preventing deaths due to substance poisoning, alcohol-related disease, and suicide.

## Supplementary Information

Below is the link to the electronic supplementary material.

Supplementary file1 (DOCX 48 KB)

## Data Availability

Data used in this study were provided by data custodians under strict restrictions for storage and use. Accordingly, the datasets are not publicly available or able to be provided by the authors.
