# Supplementary material for: Age, period, and cohort trends of substance poisoning, alcohol-related disease, and suicide deaths in Australia, 1980–2019
Source: Soc Psychiatry Psychiatr Epidemiol. 2024 Aug 23;60(3):693–703. doi: 10.1007/s00127-024-02749-4 (PMC11870947; doi:10.1007/s00127-024-02749-4)
Supplement: Supplementary file 1 — Supplementary file1 (DOCX 48 KB) [file 127_2024_2749_MOESM1_ESM.docx]

**Age, period, and cohort trends of substance poisoning, alcohol-related disease, and suicide deaths in Australia, 1980-2019**

**Appendix**

**Contents**

[Appendix Table 1. International Classification of Diseases code definitions for defining deaths due to substance poisoning, alcohol-related disease, and suicide 2](#_Toc145940064)

[Appendix Table 2. Age-standardized mortality rates (per 100,000 person years) for suicide, alcohol-related disease, and substance poisoning, 1980-2019, for Australians aged 15 years and above 3](#_Toc145940065)

[Appendix Table 3. Age-standardised mortality rates (per 100,000 person years) for suicide, alcohol-related disease, and substance poisoning, 1980-2019, Australians aged 15 years and above 4](#_Toc145940066)

[Appendix Table 4. Net drift estimates and their 95% confidence intervals (ages 20 - 90) 5](#_Toc145940067)

# Appendix Table 1. International Classification of Diseases code definitions for defining deaths due to substance poisoning, alcohol-related disease, and suicide

| Cause of death | ICD-9 codes | ICD-10 codes |
| --- | --- | --- |
| Substance poisoning (excluding intentional overdose) | 292  304  305.2-305.9  E850-E858  E980.0-E980.5  291.0-291.5  291.8  291.9  303.0  305.0  303.9  E860.0-E860.4  E860.8  E860.9 | X40-X45  Y10-Y15  Y45  Y47  Y49  F10-F16  F18  F19 |
| Alcohol-related disease | 357.5  425.5  535.3  571.0-571.3  571.4-571.9 | K70  K73  K74  E24.4  G31.2  G62.1  G72.1  I42.6  K29.2  K85.2  K86.0 |
| Suicide | E950-E959 | X60-X69  X70-X79  X80-X84  Y87 |

ICD-9: International Classification of Diseases – 9^th^ Revision; ICD-10: International Classification of Diseases – 10^th^ Revision

# Appendix Table 2. Age-standardized mortality rates (per 100,000 person years) for suicide, alcohol-related disease, and substance poisoning, 1980-2019, for Australians aged 15 years and above

| Year | Combined | Alcohol-related disease | Substance poisoning | Suicide |
| --- | --- | --- | --- | --- |
| 1980 | 28.364 | 10.857 | 2.177 | 15.330 |
| 1981 | 27.706 | 10.192 | 1.948 | 15.566 |
| 1982 | 28.208 | 10.757 | 1.941 | 15.510 |
| 1983 | 26.458 | 9.210 | 2.581 | 14.667 |
| 1984 | 27.532 | 9.744 | 2.981 | 14.807 |
| 1985 | 28.338 | 9.811 | 3.230 | 15.297 |
| 1986 | 28.668 | 9.658 | 2.450 | 16.560 |
| 1987 | 30.143 | 9.917 | 2.942 | 17.284 |
| 1988 | 30.366 | 9.664 | 3.857 | 16.845 |
| 1989 | 28.605 | 9.525 | 3.430 | 15.650 |
| 1990 | 29.063 | 9.128 | 3.541 | 16.394 |
| 1991 | 28.778 | 8.712 | 3.194 | 16.872 |
| 1992 | 28.823 | 8.516 | 4.012 | 16.295 |
| 1993 | 26.347 | 7.864 | 3.886 | 14.597 |
| 1994 | 29.359 | 8.297 | 4.787 | 16.275 |
| 1995 | 30.411 | 8.223 | 5.648 | 16.540 |
| 1996 | 29.747 | 7.883 | 5.198 | 16.666 |
| 1997 | 31.029 | 8.181 | 4.868 | 17.980 |
| 1998 | 32.040 | 7.274 | 7.039 | 17.727 |
| 1999 | 32.973 | 7.602 | 8.815 | 16.556 |
| 2000 | 29.739 | 6.868 | 7.082 | 15.789 |
| 2001 | 26.844 | 6.549 | 4.186 | 16.109 |
| 2002 | 26.214 | 7.422 | 3.810 | 14.982 |
| 2003 | 24.620 | 6.675 | 4.248 | 13.697 |
| 2004 | 24.249 | 6.568 | 4.358 | 13.323 |
| 2005 | 24.082 | 6.676 | 4.454 | 12.952 |
| 2006 | 23.753 | 6.495 | 4.251 | 13.007 |
| 2007 | 25.343 | 6.671 | 5.145 | 13.527 |
| 2008 | 26.124 | 6.685 | 5.782 | 13.657 |
| 2009 | 26.517 | 6.498 | 6.464 | 13.555 |
| 2010 | 25.761 | 6.095 | 6.085 | 13.581 |
| 2011 | 25.887 | 5.985 | 6.249 | 13.653 |
| 2012 | 25.565 | 5.753 | 5.835 | 13.977 |
| 2013 | 26.125 | 6.083 | 5.927 | 14.115 |
| 2014 | 28.537 | 6.469 | 6.836 | 15.232 |
| 2015 | 29.559 | 6.415 | 7.175 | 15.969 |
| 2016 | 28.763 | 6.172 | 7.573 | 15.018 |
| 2017 | 31.187 | 6.664 | 7.717 | 16.806 |
| 2018 | 29.393 | 5.807 | 7.831 | 15.755 |
| 2019 | 29.868 | 6.180 | 7.506 | 16.182 |

# Appendix Table 3. Age-standardised mortality rates (per 100,000 person years) for suicide, alcohol-related disease, and substance poisoning, 1980-2019, Australians aged 15 years and above

| Year | Females | | | | Males | | | |
| --- | --- | --- | --- | --- | --- | --- | --- | --- |
|  | **Combined** | **Alcohol-related disease** | **Substance poisoning** | **Suicide** | **Combined** | **Alcohol-related disease** | **Substance poisoning** | **Suicide** |
| 1980 | 14.576 | 4.043 | 2.142 | 7.774 | 43.519 | 18.083 | 2.221 | 23.214 |
| 1981 | 14.308 | 3.921 | 1.645 | 8.055 | 42.573 | 16.849 | 2.244 | 23.479 |
| 1982 | 15.293 | 4.673 | 1.735 | 8.086 | 42.672 | 17.231 | 2.148 | 23.293 |
| 1983 | 13.426 | 3.905 | 1.949 | 7.008 | 40.812 | 14.856 | 3.223 | 22.733 |
| 1984 | 13.688 | 3.552 | 2.398 | 7.024 | 42.820 | 16.318 | 3.580 | 22.922 |
| 1985 | 14.395 | 3.817 | 2.526 | 7.067 | 43.954 | 16.192 | 3.940 | 23.822 |
| 1986 | 14.081 | 4.017 | 1.809 | 7.602 | 44.569 | 15.599 | 3.091 | 25.878 |
| 1987 | 13.486 | 3.709 | 1.902 | 6.987 | 48.456 | 16.474 | 4.007 | 27.975 |
| 1988 | 14.547 | 3.610 | 2.597 | 7.277 | 47.961 | 16.068 | 5.137 | 26.757 |
| 1989 | 13.243 | 3.646 | 2.144 | 6.516 | 45.605 | 15.763 | 4.727 | 25.115 |
| 1990 | 13.118 | 3.478 | 2.070 | 6.666 | 46.552 | 15.074 | 5.024 | 26.454 |
| 1991 | 13.296 | 3.371 | 2.151 | 7.113 | 45.580 | 14.341 | 4.265 | 26.974 |
| 1992 | 12.526 | 2.746 | 2.140 | 6.754 | 46.653 | 14.586 | 5.921 | 26.146 |
| 1993 | 12.026 | 3.338 | 2.285 | 5.681 | 41.939 | 12.601 | 5.515 | 23.823 |
| 1994 | 13.079 | 3.607 | 2.783 | 5.939 | 47.027 | 13.256 | 6.827 | 26.945 |
| 1995 | 13.801 | 3.386 | 2.680 | 7.094 | 48.261 | 13.296 | 8.681 | 26.284 |
| 1996 | 12.332 | 3.204 | 2.416 | 6.212 | 48.285 | 12.766 | 8.044 | 27.475 |
| 1997 | 14.195 | 3.206 | 2.789 | 7.500 | 49.178 | 13.370 | 6.999 | 28.809 |
| 1998 | 14.285 | 3.169 | 3.360 | 6.964 | 51.203 | 11.544 | 10.827 | 28.831 |
| 1999 | 14.833 | 3.401 | 4.536 | 6.399 | 52.247 | 11.964 | 13.225 | 27.057 |
| 2000 | 13.825 | 2.817 | 3.875 | 6.577 | 46.766 | 11.068 | 10.390 | 25.308 |
| 2001 | 12.550 | 2.403 | 2.801 | 6.643 | 42.343 | 10.835 | 5.617 | 25.892 |
| 2002 | 12.818 | 3.365 | 2.511 | 6.388 | 40.596 | 11.602 | 5.149 | 23.845 |
| 2003 | 11.982 | 2.638 | 2.841 | 5.852 | 38.314 | 10.837 | 5.699 | 21.777 |
| 2004 | 11.618 | 2.707 | 2.770 | 5.493 | 37.910 | 10.539 | 5.998 | 21.373 |
| 2005 | 11.904 | 3.086 | 2.945 | 5.344 | 37.147 | 10.369 | 6.006 | 20.772 |
| 2006 | 12.644 | 2.909 | 3.013 | 6.001 | 35.897 | 10.167 | 5.527 | 20.203 |
| 2007 | 13.996 | 3.258 | 3.717 | 6.360 | 37.621 | 10.160 | 6.609 | 20.852 |
| 2008 | 13.120 | 3.192 | 3.655 | 5.687 | 40.016 | 10.258 | 7.965 | 21.794 |
| 2009 | 14.397 | 3.208 | 4.251 | 6.342 | 39.494 | 9.858 | 8.727 | 20.908 |
| 2010 | 13.524 | 3.040 | 3.803 | 6.155 | 38.803 | 9.218 | 8.434 | 21.151 |
| 2011 | 14.129 | 3.216 | 3.752 | 6.584 | 38.448 | 8.815 | 8.808 | 20.825 |
| 2012 | 14.132 | 2.750 | 4.102 | 6.788 | 37.731 | 8.817 | 7.614 | 21.300 |
| 2013 | 14.358 | 2.804 | 3.921 | 7.116 | 38.639 | 9.420 | 7.998 | 21.221 |
| 2014 | 15.110 | 3.037 | 4.242 | 7.272 | 42.882 | 9.989 | 9.523 | 23.369 |
| 2015 | 16.202 | 3.340 | 4.366 | 7.869 | 43.887 | 9.553 | 10.094 | 24.240 |
| 2016 | 15.920 | 3.253 | 4.633 | 7.439 | 42.567 | 9.165 | 10.636 | 22.766 |
| 2017 | 16.658 | 3.237 | 4.594 | 8.241 | 46.730 | 10.167 | 10.972 | 25.591 |
| 2018 | 15.768 | 2.946 | 4.711 | 7.539 | 44.051 | 8.757 | 11.093 | 24.201 |
| 2019 | 16.235 | 3.217 | 4.679 | 7.739 | 44.560 | 9.222 | 10.469 | 24.869 |

# Appendix Table 4. Net drift estimates and their 95% confidence intervals (ages 20 - 90)

|  | Net drift  (95% CI) | Min local drift  (95% CI) | Max local drift  (95% CI) |
| --- | --- | --- | --- |
| Females | 0.42 (0.17, 0.68) | -0.85 (-1.05, -0.65) at 68 | 1.89 (0.55, 3.24) at 90 |
| Alcohol-related disease | -0.28 (-0.66, 0.09) | -1.37 (-1.64, -1.09) at 66 | 3.15 (0.56, 5.80) at 90 |
| Substance poisoning | 2.58 (2.18, 2.98) | -3.08 (-3.68, -2.48) at 20 | 4.51 (4.14, 4.89) at 48 |
| Suicide | -0.25 (-0.52, 0.01) | -1.39 (-1.04, -0.25) at 76 | 0.43 (0.22, 0.63) at 42 |
| Males | -0.41 (-0.59, -0.24) | -1.67 (-1.80, -1.55) at 68 | 1.08 (0.98, 1.18) at 42 |
| Alcohol-related disease | -1.46 (-1.75, -1.16) | -2.93 (-4.01, -1.84) at 22 | 2.53 (0.40, 4.71) at 90 |
| Substance poisoning | 3.33 (2.84, 3.83) | -2.53 (-2.92, -2.14) at 20 | 7.55 (7.13, 7.98) at 46 |
| Suicide | -0.52 (-0.69, -0.36) | -1.51 (-1.76, -1.27) at 78 | 0.81 (0.69, 0.92) at 44 |
